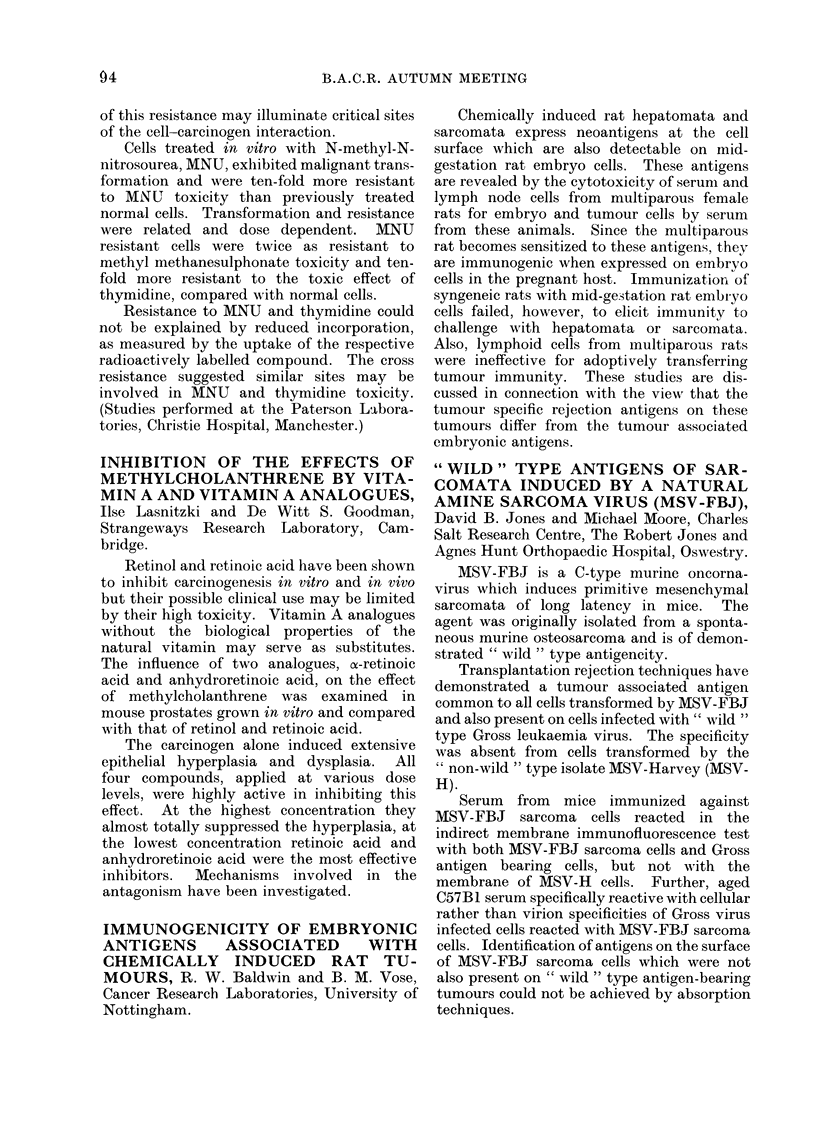# Proceedings: Inhibition of the effects of methylcholanthrene by vitamin A and vitamin A analogues.

**DOI:** 10.1038/bjc.1974.20

**Published:** 1974-01

**Authors:** I. Lasnitzki, D. S. Goodman


**INHIBITION OF THE EFFECTS OF METHYLCHOLANTHRENE BY VITAMIN A AND VITAMIN A ANALOGUES,** Ilse Lasnitzki and De Witt S. Goodman, Strangeways Research Laboratory, Cambridge.

Retinol and retinoic acid have been shown to inhibit carcinogenesis *in vitro* and *in vivo* but their possible clinical use may be limited by their high toxicity. Vitamin A analogues without the biological properties of the natural vitamin may serve as substitutes. The influence of two analogues, α-retinoic acid and anhydroretinoic acid, on the effect of methylcholanthrene was examined in mouse prostates grown *in vitro* and compared with that of retinol and retinoic acid.

The carcinogen alone induced extensive epithelial hyperplasia and dysplasia. All four compounds, applied at various dose levels, were highly active in inhibiting this effect. At the highest concentration they almost totally suppressed the hyperplasia, at the lowest concentration retinoic acid and anhydroretinoic acid were the most effective inhibitors. Mechanisms involved in the antagonism have been investigated.